# Association Between Social Media Use Duration and Depressive and Anxiety Symptoms in Adolescents: A Retrospective Study

**DOI:** 10.62641/aep.v54i4.2228

**Published:** 2026-08-15

**Authors:** Lanlan Feng, Zhaojun Tan

**Affiliations:** ^1^Department of Sleep Disorders and Neurosis, The Second People’s Hospital of Hunan Province (Brain Hospital of Hunan Province), 410007 Changsha, Hunan, China

**Keywords:** adolescent, depression, social media, anxiety

## Abstract

**Background:**

This study aimed to investigate the relationship between the duration of social media use and the prevalence and severity of depressive and anxiety symptoms among adolescents in the real-world settings, thereby providing data support to inform mental health support and guide appropriate social media use.

**Methods:**

This retrospective study analysed clinical data from 156 adolescents admitted to the Mental Health Service Centre of The Second People’s Hospital of Hunan Province (Brain Hospital of Hunan Province) between January 2022 and December 2023. Data were extracted from electronic medical record system and structured survey forms. Baseline demographic characteristics, social media use related data, and mental health assessment results were collected. Pearson correlation analysis was used to examine relationship between social media use duration and scores on the 9-item Patient Health Questionnaire (PHQ-9) and 7-item Generalized Anxiety Disorder scale (GAD-7). Multivariate logistic regression was performed to identify factors independently associated with depression and anxiety symptoms.

**Results:**

Univariate analysis revealed statistically significant differences in parenting style, academic performance, weekly exercise frequency, and depression and anxiety symptoms across different durations of social media use (*p* < 0.05). Correlation analysis demonstrated significant positive correlations between average daily social media use duration and PHQ-9 score (r = 0.352, *p* < 0.001) and GAD-7 score (r = 0.308, *p* < 0.001). Multivariate logistic regression identified longer social media use as an independent risk factor for positive depressive symptoms (odds ratio (OR) = 1.794, 95% confidence interval (CI): 1.261–2.547), along with authoritative parenting (OR = 2.485, 95% CI: 1.253–4.922), permissive parenting (OR = 3.427, 95% CI: 1.503–7.816), medium academic performance (OR = 2.867, 95% CI: 1.286–6.396), and poor academic performance (OR = 6.118, 95% CI: 2.534–14.779). For positive anxiety symptoms, independent risk factors included longer social media use (OR = 1.624, 95% CI: 1.169–2.267), authoritative parenting (OR = 2.278, 95% CI: 1.171–4.425), permissive parenting (OR = 2.863, 95% CI: 1.317–6.256), medium academic performance (OR = 2.564, 95% CI: 1.176–5.614), poor academic performance (OR = 4.575, 95% CI: 1.975–10.614), and reduced weekly exercise frequency (1–2 times/week: OR = 1.927, 95% CI: 1.027–3.608; <1 time/week: OR = 3.825, 95% CI: 1.896–7.736).

**Conclusions:**

Social media use duration is significantly associated with depressive and anxiety symptoms in adolescents. These findings underscore the importance of monitoring adolescent social media use and implementing targeted health guidance to reduce mental health risks.

## Introduction

With the widespread adoption of digital technology, social media has become a central platform for adolescents’ social interaction, information seeking and self-expression. The prevalence and daily usage duration of social media among adolescents have continued to increase [1]. A substantial proportion of adolescents spend several hours per day on social media platforms [2]. While social media breaks the limitations of time and space and expands the boundaries of social interaction, the problems it brings, such as information overload, virtual social dependence and sleep deprivation, have also gradually attracted attention. In particular, adolescents are in a critical developmental period characterized by ongoing brain maturation, self-identity formation and the acquisition of emotional skills. Their psychological resilience is relatively limited, and excessive exposure to the social media environment may confer unique mental health risks. In recent years, the global prevalence of depressive and anxiety symptoms among adolescents has risen markedly, with depression- and anxiety-related mood disorders accounting for over 60%, representing a major public health concern that adversely affects adolescents’ physical health, psychological well-being and academic development [3]. In this context, numerous researchers have investigated the potential link between social media use and adolescent mental health. Studies have shown that excessive social media use may be related to social comparison, cyberbullying, sleep deprivation and social withdrawal, which may in turn be associated with depression and anxiety symptoms [4, 5]. Cross-sectional study have suggested that associations between social media use and negative emotions in adolescents [6]; however, such studies predominantly reply on single time-point analyses and often adequately control for confounding factors such as parenting style, academic pressure and exercise habits, resulting in inconsistent findings. Furthermore, most existing studies rely on the self-reported questionnaires data collected under idealized conditions, which may not accurately reflect the complexity and diversity of real-world social media use among adolescents, thereby limiting the ability to establish precise dose-effect relationship between usage duration and depression and anxiety symptoms.

Based on this, this study analysed data from 156 adolescents recruited from Mental Health Service Centres. Through retrospective analysis of clinical records and visit evaluation data, this study aimed to elucidate the association between social media usage durations and the presence and severity of depressive and anxiety symptoms. Social media includes commonly used platforms such as WeChat (v8.0.x, Tencent Inc., Shenzhen, China), TikTok Chinese version/Douyin (v22.x–v28.x, Beijing Microlive Vision Technology Co., Ltd., Beijing, China) and QQ (v9.0.x, Tencent Inc., Shenzhen, China). It also incorporated potential confounding factors such as parenting styles, academic performance and exercise frequency for multidimensional analysis. The aim was to determine whether excessive social media use is independently associated with depressive and anxiety symptoms in adolescents, to identify clinical meaningful usage duration thresholds, and to provide real-world evidence to develop scientific norms for adolescent social media use and conduct precise mental health supports. Ultimately, these efforts may contribute to the development of a collaborative adolescent mental health protection system involving families, schools and society.

## Materials and Methods

### General Information

A total of 156 adolescents who underwent psychological screening at the Mental Health Service Centre of The Second People’s Hospital of Hunan Province (Brain Hospital of Hunan Province) between January 2022 and December 2023 were enrolled. The research ethics approval number is HNUCN11-2409-380, and this study strictly followed the Declaration of Helsinki [7]. Written informed consent was obtained from all participants and their guardians. All participants were recruited from the Mental Health Service Centre of The Second People’s Hospital of Hunan Province, representing a help-seeking clinical subgroup rather than the general adolescent population. The inclusion criteria were as follows: (1) age 12–18 years old; (2) availability of health records documenting average daily social media use (hours/day, self-reported by adolescents and confirmed by the parents), the main platform types (e.g., WeChat, QQ, TikTok Chinese version/Douyin) during the exposure assessment period; and (3) at least one complete mental health assessment record between January 2022 and December 2023. The exclusion criteria were as follows: (1) previous diagnosis of schizophrenia or bipolar disorder; or (2) presence of any medical condition that could affect mental health assessment results; Of 182 adolescents were assessed; 26 were excluded (15 due to incomplete medical records; 11 due to a history of schizophrenia, bipolar disorder, or current major physical illness). Finally, 156 participants were included. The exposure assessment period was defined as the year 2021, during which social media use data were extracted from electronic health records.

### Survey Tools

Data was collected through a combination of electronic medical record review and structured survey form. The collected information included: sex, age, grade, family structure, parental education level, only child status, parenting style, boarding school status, academic performance and weekly physical activity frequency. Average daily social media use duration was obtained from health records, as reported by adolescents and confirmed by parents; these data reflected usage over the past month, with weekday and weekend usage were not assessed separately. These individual questions were not standardized psychological scales; therefore, reliability and validity tests were not calculated. Mental health were assessed using the 9-item Patient Health Questionnaire (PHQ-9) (Cronbach’s α = 0.86–0.89) to assess depressive symptoms, with a score of ≥5 indicating the presence of depressive symptoms [8]; the 7-item Generalized Anxiety Disorder scale (GAD-7) (Cronbach’s α = 0.91–0.92) was used to assess anxiety symptoms, with a score of ≥5 indicating the presence of anxiety symptoms [9]. The outcome measures (PHQ-9, GAD-7) data were extracted from the evaluation records completed during visits to the Mental Health Service Centre between January 2022 and December 2023. Family structure was categorized into two groups: nuclear family and non-nuclear family. A nuclear family was defined as a household consisting of two parents (married or cohabiting) and their dependent children, with no extended family members residing in the home. Non-nuclear family included all other household configurations, such as single-parent families, multigenerational, blended families, and households with non-parental guardians [10]. Parenting style was self-reported by adolescent participants based on their perceptions of their parents’ daily behaviours and was classified into three types: democratic, authoritative and permissive. Democratic parenting was characterized by open communication, mutual respect and collaborative decision-making. Authoritative parenting was defined by rigid rules, punishment-oriented discipline, blaming children for mistakes, closed communication and an autocratic environment. Permissive parenting was defined by high warmth and affection but low behavioural control, with minimal rule enforcement and few demands on children [11]. This study did not use a standardized parenting style scale, and the classification was based on participants’ self-reported choices. Academic performance was assessed using a single question: “How do you evaluate your academic performance compared with other students in your class/grade?” with response options of “excellent” (top of the class), “good” (above average), “medium” (average level), “poor” (middle or lower level). This assessment was based on participants’ subjective self-report. All data were derived from existing electronic medical records; not active follow-up was conducted.

### Statistical Methods

Data were analysed using SPSS 26.0 (IBM Corp., Armonk, NY, USA) statistical software. Normality tests were performed. Normally distributed data are presented as mean ± standard deviation ($\bar{x}$ ± s). Between-group comparisons were performed using independent samples *t*-test. Count data were presented as number (n [%]) and were compared using χ^2^ test, with Cramér’s V calculated as a measure of effect size. Pearson correlation analysis was used to examine the relationships between social media use duration and PHQ-9 and GAD-7 score. Multivariate Logistic regression analyses were performed to identify independent influencing factors for positive depressive symptoms and positive anxiety symptoms in adolescents. All tests were two-sided, and a significance level of α = 0.05 was used.

## Results

### Univariate Analysis of Adolescents’ Social Media Usage Time

In the univariate analysis of positive depression/anxiety symptoms, statistically significant differences were observed in parental parenting styles, academic performance, weekly exercise frequency, and duration of social media usage (*p* < 0.05, Table 1).

**Table 1.** **Univariate analysis comparing adolescents with and without positive depressive/anxiety symptoms**.

Variable		Positive for depressive symptoms (n = 65)	Negative for depressive symptoms (n = 91)	*t*/χ^2^	*p*	Cramér’s V	Positive for anxiety symptoms (n = 57)	Negative for anxiety symptoms (n = 99)	*t*/χ^2^	*p*	Cramér’s V
Gender				0.018	0.892	0.011			0.011	0.918	0.008
	Male	30 (46.2)	43 (47.3)				26 (45.6)	46 (46.5)			
	Female	35 (53.8)	48 (52.7)				31 (54.4)	53 (53.5)			
Age		15.31 ± 1.90	15.22 ± 1.84	0.290	0.772	–	15.39 ± 2.03	15.29 ± 1.92	0.285	0.776	–
Family structure				0.014	0.904	0.009			0.001	0.977	0.003
	Nuclear family	47 (72.3)	65 (71.4)				41 (71.9)	71 (71.7)			
	Non-nuclear family	18 (27.7)	26 (28.6)				16 (28.1)	28 (28.3)			
Parents’ education level				0.178	0.673	0.034			0.077	0.781	0.022
	High school and above	40 (61.5)	59 (64.8)				35 (61.4)	63 (63.6)			
	Below high school	25 (38.5)	32 (35.2)				22 (38.6)	36 (36.4)			
Only child				0.001	0.978	0.003			0.026	0.872	0.013
	Yes	37 (56.9)	52 (57.1)				33 (57.9)	56 (56.6)			
	No	28 (43.1)	39 (42.9)				24 (42.1)	43 (43.4)			
Parenting style				6.097	0.047	0.198			6.736	0.034	0.208
	Democratic	25 (38.5)	53 (58.2)				20 (35.1)	56 (56.6)			
	Authoritative	28 (43.1)	25 (27.5)				25 (43.9)	28 (28.3)			
	Permissive	12 (18.5)	13 (14.3)				12 (21.0)	15 (15.1)			
Boarding				0.034	0.855	0.015			0.060	0.806	0.020
	Yes	18 (27.7)	24 (26.4)				16 (28.1)	26 (26.3)			
	No	47 (72.3)	67 (73.6)				41 (71.9)	73 (73.7)			
Academic performance				21.597	<0.001	0.372			11.484	0.009	0.271
	Excellent	5 (7.7)	23 (25.3)				6 (10.5)	22 (22.2)			
	Good	17 (26.2)	40 (44.0)				15 (26.3)	42 (42.4)			
	Medium	20 (30.8)	17 (18.7)				19 (33.3)	18 (18.2)			
	Poor	23 (35.4)	11 (12.1)				17 (29.8)	17 (17.2)			
Weekly exercise frequency				45.771	<0.001	0.542			15.644	<0.001	0.317
	≥ 3 times	6 (9.2)	38 (41.8)				8 (14.0)	36 (36.4)			
	1–2 times	20 (30.8)	43 (47.3)				21 (36.8)	42 (42.4)			
	< 1 time	39 (60.0)	10 (11.0)				28 (49.1)	21 (21.2)			
Social media usage duration (hours/day)		4.82 ± 2.14	2.74 ± 1.17	7.795	<0.001	–	4.63 ± 2.08	2.97 ± 1.37	5.390	<0.001	–
Total score of PHQ-9		10.26 ± 3.54	3.14 ± 1.20	17.840	<0.001	–	–	–			
Total score of GAD-7		–	–				9.86 ± 3.30	3.56 ± 1.55	13.598	<0.001	–

PHQ-9, 9-item Patient Health Questionnaire; GAD-7, 7-item Generalized Anxiety Disorder scale; χ^2^, chi-square test; Cramér’s V, Cramér’s V (effect size for chi-square test).

### Correlation Analysis Between Adolescents’ Social Media Usage Time and Symptoms of Depression and Anxiety

The correlation analysis results showed that the adolescents’ average daily social media use duration was significantly positively correlated with the PHQ-9 score (r = 0.352, *p* < 0.001), indicating that longer the social media use duration, the higher depression score. Similarly, social media use duration was significantly positively correlated with the GAD-7 score (r = 0.308, *p* < 0.001, Table 2).

**Table 2.** **Correlation analysis between adolescents’ social media usage time and symptoms of depression and anxiety**.

Variable	r value	*p* value
Social media usage time and PHQ-9 score	0.352	< 0.001
Social media usage time and GAD-7 score	0.308	< 0.001

### Multivariate Analysis of Adolescents’ Social Media Usage Time

Regression analysis was performed using positive depressive symptoms and anxiety symptoms as dependent variables, and variables that were statistically significant in univariate analysis were included. Variable assignments are shown in Table 3. For depressive symptoms, each additional hour of daily social media use was associated with an odds ratio (OR) of 1.794 (95% confidence interval (CI): 1.261–2.547). Compared with democratic parenting, authoritative parenting (OR = 2.485, 95% CI: 1.253–4.922) and permissive parenting (OR = 3.427, 95% CI: 1.503–7.816) were associated with higher odds of depressive symptoms. For academic performance, compared with excellent performance, medium performance (OR = 2.867, 95% CI: 1.286–6.396) and poor performance (OR = 6.118, 95% CI: 2.534–14.779) were associated with significantly higher odds of depressive symptoms. Weekly exercise frequency was not independently associated with depressive symptoms after adjusting for other covariates (*p* > 0.05); therefore, it was not retained in the final model. The corresponding regression results are not presented in Table 4.

**Table 3.** **Variable assignment table**.

Project	Variable type	Coding/categories	Reference category
Depressive symptoms	Binary	0 = Negative (PHQ-9 < 5)	
		1 = Positive (PHQ-9 ≥ 5)	
Anxiety symptoms	Binary	0 = Negative (GAD-7 < 5)	
		1 = Positive (GAD-7 ≥ 5)	
Social media usage duration	Continuous	Original value	
Parenting style	Categorical	1 = Democratic	Democratic
		2 = Authoritative	
		3 = Permissive	
Academic performance	Categorical	1 = Excellent	Excellent
		2 = Good	
		3 = Medium	
		4 = Poor	
Weekly exercise frequency	Categorical	1 = ≥ 3 times/week	≥ 3 times/week
		2 = 1–2 times/week	
		3 = < 1 time/week	

PHQ-9, 9-item Patient Health Questionnaire; GAD-7, 7-item Generalized Anxiety Disorder scale.

**Table 4.** **Multivariate logistic regression analysis of factors associated with depressive symptoms in adolescents**.

Project		β	SE	Wald	*p*	OR	95% CI
Social media usage time		0.587	0.182	10.376	0.001	1.794	1.261–2.547
Parenting style (Ref: democratic)							
	Authoritative	0.912	0.353	6.761	0.009	2.485	1.253–4.922
	Permissive	1.236	0.427	8.576	0.003	3.427	1.503–7.816
Academic performance (Ref: excellent)							
	Good	0.453	0.397	1.332	0.249	1.574	0.735–3.367
	Medium	1.053	0.413	6.562	0.010	2.867	1.286–6.396
	Poor	1.815	0.452		<0.001	6.118	2.534–14.779
Constant		–3.50	0.654	28.963	<0.001	0.003	

OR, odds ratio; CI, confidence interval; SE, standard error; Ref, reference category. Weekly exercise frequency was not statistically significant (*p* > 0.05) in multivariate analysis and thus not retained.

For anxiety symptoms, each additional hour of daily social media use was associated with an OR of 1.624 (95% CI: 1.169–2.267). Compared with democratic parenting, authoritative parenting (OR = 2.278, 95% CI: 1.171–4.425) and permissive parenting (OR = 2.863, 95% CI: 1.317–6.256) were associated with higher odds. For academic performance, compared with excellent performance, medium performance (OR = 2.564, 95% CI: 1.176–5.614) and poor performance (OR = 4.575, 95% CI: 1.975–10.614) were associated with significantly higher odds of anxiety symptoms. For weekly exercise frequency, compared with ≥3 times/week, 1–2 times/week (OR = 1.927, 95% CI: 1.027–3.608) and <1 time/week (OR = 3.825, 95% CI: 1.896–7.736) were associated with progressively higher odds of anxiety symptoms (Table 5), all *p* < 0.05 (Tables 4 and 5).

**Table 5.** **Multivariate logistic regression analysis of factors associated with anxiety symptoms in adolescents**.

Project		β	SE	Wald	*p*	OR	95% CI
Social media usage time		0.487	0.173	7.975	0.005	1.624	1.169–2.267
Parenting style (Ref: democratic)							
	Authoritative	0.823	0.342	5.816	0.016	2.278	1.171–4.425
	Permissive	1.056	0.402	6.882	0.009	2.863	1.317–6.256
Academic performance (Ref: excellent)							
	Good	0.387	0.386	1.002	0.317	1.468	0.695–3.086
	Medium	0.943	0.040	5.527	0.019	2.564	1.176–5.614
	Poor	1.528	0.438	12.503	<0.001	4.575	1.975–10.614
Weekly exercise frequency (Ref: ≥ 3 times/week)							
	1–2 times/week	0.653	0.327	4.137	0.042	1.927	1.027–3.608
	< 1 time/week	1.346	0.365	13.846	<0.001	3.825	1.896–7.736
Constant		–3.20	0.62	26.634	<0.001	0.041	

OR, odds ratio; CI, confidence interval; SE, standard error; Ref, reference category.

## Discussion

This retrospective study of 156 adolescents examined the association between social media use duration and depressive and anxiety symptoms. It also clarified the influence of factors such as parenting style, academic performance and weekly exercise frequency. The findings important practical evidence to inform clinical mental health support for adolescents. Univariate analysis revealed significant differences between adolescents with and without depressive/anxiety symptoms in terms of parenting style, academic performance, weekly exercise frequency and daily social media use duration. Correlation analysis further confirmed a significant positive correlation between social media use duration and depressive and anxiety symptoms, consistent with the conclusions of most previous studies [12, 13], indicating that excessive social media use is indeed an important associated factor in adolescent mental health problems. Multivariate logistic regression analysis identified independent associated factors: social media use duration, parenting style and academic performance were independently associated with depressive symptoms, while social media use duration, academic performance and weekly exercise frequency were key associated factors for anxiety symptoms. These findings highlight the potential importance of addressing excessive social media use for adolescent mental health.

The effect of social media use duration on depressive and anxiety symptoms may be explained by several mechanisms. Firstly, prolonged social media use duration may reduce adolescents’ time for offline social interaction, physical exercise and adequate sleep. Face-to-face social interaction is essential for building supportive networks and improve mood regulation. Physical exercise has been linked to improved mood by promoting the secretion of neurotransmitters (e.g., dopamine and serotonin). Insufficient sleep may affect the brain’s mood regulation function. The lack of these key activities together provides opportunities for the occurrence of depressive and anxiety symptoms [14, 15]. Secondly, the information dissemination characteristics of social media platforms may also exacerbate psychological stress. Adolescents are easily exposed to negative information such as overly romanticized life scenes, competitive content and cyberbullying during use. This content may distort their self-perception, causing unnecessary psychological discrepancy and social anxiety. Adolescents’ psychological development is not yet mature, and their self-identity and mood regulation abilities are weak, making it difficult for them to effectively cope with such negative stimuli. Long-term accumulation may induce depression and anxiety [16, 17]. Thirdly, excessive immersion in virtual social scenarios may impair adolescents’ real-world social skills, increasing feelings of helplessness and frustration when facing interpersonal conflicts and life stressors. This may lead to further escapism through social media addiction, creating a vicious cycle of deteriorating mental health [18, 19]. From the perspective of social cognition and developmental psychology, adolescents is a critical period for self-identity construction, and the way social media content is presented often reinforces the social comparison mechanism [20]. When adolescents browse others’ carefully edited life displays, they may experience “relative deprivation” or “self-worth doubt”, especially when there is pressure in their studies, social life, or family relationships in real life, this comparison effect will be more significant [21, 22]. Prolonged immersive use not only takes away their time for real-world social interaction, outdoor sports and creative activities, but may also change their neural mechanisms of attention allocation and emotional regulation [23]. Neuroimaging studies have suggested that excessive social media use is associated with abnormal activation of the prefrontal cortex and default mode network, which may affect adolescents’ executive function, emotional regulation and social cognition, thus providing a physiological basis for the development of depressive and anxiety symptoms [24]. Therefore, this study should not only regard social media use as a behavioural problem, but also understand its effect from multiple levels (e.g., neurodevelopment, psychosocial adaptation and ecological environment). Parents’ parenting style also affects adolescents’ psychology. Democratic parenting can provide sufficient emotional support and autonomy, helping adolescents to establish a good emotional regulation mechanism, while authoritarian or permissive parenting is prone to parent–child communication barriers, making it impossible for adolescents to release negative emotions in time, and thus escape reality through excessive social media using [25, 26]. The association between academic performance and depressive symptoms may stem from the frustration and self-denial caused by poor grades, while the protective effect of regular exercise on anxiety may be mediated by the release of positive neurotransmitters such as dopamine and serotonin, physical inactivity may undermine emotional regulatory capacity.

Based on these findings, managing the duration of adolescent social media use may have practical significance for maintaining mental health. However, it is important to avoid simple time limits and instead focus on scientific guidance. From the family perspective, parents should establish reasonable rules for social media use, clarify the daily usage limit, and set an example by reducing their own excessive use. They should also enrich offline family interactions through companionship and communication to reduce adolescents’ dependence on social media [27]. Schools can help adolescents recognize the harm of excessive social media use through mental health education courses, improve their media literacy and emotional regulation abilities, and increase the duration of sports activities and group activities to provide more channels for offline social interaction and emotional release [28, 29]. At the societal level, it is necessary to strengthen the supervision of social media platforms, optimize the environment for adolescents’ use, reduce the spread of harmful information, and develop more beneficial content suitable for adolescents to guide them to use social media reasonably to acquire knowledge and broaden their horizons [30]. Although this study revealed a significant association between social media use duration and depressive and anxiety symptoms, it is still necessary to pay attention to the complexity and two-way possibilities involved. On the one hand, social media use may lead to mental health problems; on the other hand, adolescents with pre-existing depression or anxiety tendencies may be more inclined to use social media as a way to escape emotions or seek validation [31, 32]. This two-way relationship suggests caution when interpreting research results to avoid simplistic attribution. Future research could further explore the differentiated effects of different content and use duration on adolescent mental health, providing a basis for developing more targeted support strategies. Ultimately, helping adolescents protect their mental health while enjoying the convenience of social media.

The strengths of this study lie in its reliance on real-world clinical and school screening data, resulting in a highly representative sample. Furthermore, the inclusion of multiple confounding factors in the analysis contributes to the high reliability of the results. However, this study has several limitations: the retrospective design may lead to recall bias in information such as social media use duration and exercise frequency; the lack of detail regarding social media content and usage duration suggests potential differences in the effect of different usage scenarios; the single-centre study design limits the extrapolation of results; and the interaction effects between various factors were not explored. In addition, variables such as duration of social media use, exercise frequency, academic performance and parenting styles are all based on self-report by adolescents, lacking objective verification, and may have recall bias and social expectation bias. Unmeasured confounders include socioeconomic status, family income, sleep duration/quality, prior psychiatric history and academic stress, which may bias the results. The assessment period social media use (past month) differs from that for PHQ-9 and GAD-7 (past two weeks), which may introduce recall bias and temporal misalignment. Future studies should harmonize assessment time frames. The temporal mismatch between exposure assessment (2021) and outcome measurements (2022–2023) may underestimate or overestimate associations if social media use changed over time. Reverse causality is possible: adolescents with existing depressive or anxiety symptoms may use social media more heavily. Due to the retrospective design, baseline symptom severity was not available for adjustment. Future prospective studies are needed. Future research could include multicentre prospective cohort studies to further validate causal relationships; refinement of social media use indicators to clarify the effect of different usage patterns; and longitudinal observational studies to explore the effects of comprehensive measures such as limiting usage time, optimizing parenting methods, and strengthening physical exercise on improving adolescent mental health. Future studies should differentiate between active and passive use, content types (e.g., entertainment vs. social comparison), and time of use (e.g., before bedtime), as these may have stronger effects on mental health than duration alone.

## Conclusions

This retrospective study found a significant positive correlation between social media use duration and depressive and anxiety symptoms among adolescents. Multivariate logistic regression identified social media use, parenting style, academic performance and weekly exercise frequency as key associated factors for adolescent mental health outcomes. These results suggest that addressing excessive social media use may be relevant to adolescent mental health. Future research should adopt longitudinal designs to further investigate causal relationships and refine analyses of specific social media use patterns.

## Availability of Data and Materials

The datasets used and/or analyzed during the current study were available from the corresponding author on reasonable request.

## References

[1] Carraturo F, Di Perna T, Giannicola V, Nacchia MA, Pepe M, Muzii B (2023). Envy, Social Comparison, and Depression on Social Networking Sites: A Systematic Review. *European Journal of Investigation in Health, Psychology and Education*.

[2] Ray N, Khan MK, Islam FN, Amin MA, Rahman ST (2025). Usage Pattern and Health Impact of Social Media on School-going Adolescents in Dhaka, Bangladesh. https://mmjmmc.com/uploads/vvGsB1mo1Nmz.htm.

[3] Robson EM, Husin HM, Ghazaleh Dashti S, Vijayakumar N, Moreno-Betancur M, Moran P (2025). Tracking the course of depressive and anxiety symptoms across adolescence (the CATS study): a population-based cohort study in Australia. *The Lancet. Psychiatry*.

[4] Houghton S, Lawrence D, Hunter SC, Rosenberg M, Zadow C, Wood L (2018). Reciprocal Relationships between Trajectories of Depressive Symptoms and Screen Media Use during Adolescence. *Journal of youth and adolescence*.

[5] Twenge MJ, Joiner TE, Rogers ML, Martin GN (2018). Increases in Depressive Symptoms, Suicide-Related Outcomes, and Suicide Rates Among U.S. Adolescents After 2010 and Links to Increased New Media Screen Time. *Clinical Psychological Science*.

[6] Primack BA, Shensa A, Sidani JE, Whaite EO, Lin LY, Rosen D (2017). Social Media Use and Perceived Social Isolation Among Young Adults in the U.S. *American Journal of Preventive Medicine*.

[7] World Medical Association (2025). World Medical Association Declaration of Helsinki: Ethical Principles for Medical Research Involving Human Participants. *JAMA*.

[8] Ganguly S, Samanta M, Roy P, Chatterjee S, Kaplan DW, Basu B (2013). Patient health questionnaire-9 as an effective tool for screening of depression among Indian adolescents. *The Journal of Adolescent Health: Official Publication of the Society for Adolescent Medicine*.

[9] Spitzer RL, Kroenke K, Williams JB, Löwe B (2006). A brief measure for assessing generalized anxiety disorder: the GAD-7. *Archives of Internal Medicine*.

[10] Huang XX, Li YT, Chen JH, Ma JJ, Cong EZ, Xu YF (2023). The influence of family structure on depression and anxiety symptoms in adolescents: the mediating role of emotional neglect. *Zhongguo dang dai er ke za zhi = Chinese journal of contemporary pediatrics*.

[11] Salavera C, Usán P, Quilez-Robres A (2022). Exploring the Effect of Parental Styles on Social Skills: The Mediating Role of Affects. *International Journal of Environmental Research and Public Health*.

[12] Saleem SM, Jan SS (2024). A cross-sectional study of mental health effects of excessive screen time and social media use among Indian adolescents and young adults. *Journal of Nature and Science of Medicine*.

[13] Frielingsdorf H, Fomichov V, Rystedt I, Lindstrand S, Korhonen L, Henriksson H (2025). Associations of time spent on different types of digital media with self-rated general and mental health in Swedish adolescents. *Scientific Reports*.

[14] Vannucci A, Flannery KM, Ohannessian CM (2017). Social media use and anxiety in emerging adults. *Journal of Affective Disorders*.

[15] Oberst U, Wegmann E, Stodt B, Brand M, Chamarro A (2017). Negative consequences from heavy social networking in adolescents: The mediating role of fear of missing out. *Journal of Adolescence*.

[16] Seabrook EM, Kern ML, Rickard NS (2016). Social Networking Sites, Depression, and Anxiety: A Systematic Review. *JMIR Mental Health*.

[17] Nesi J, Prinstein MJ (2015). Using Social Media for Social Comparison and Feedback-Seeking: Gender and Popularity Moderate Associations with Depressive Symptoms. *Journal of Abnormal Child Psychology*.

[18] Sampasa-Kanyinga H, Lewis RF (2015). Frequent Use of Social Networking Sites Is Associated with Poor Psychological Functioning Among Children and Adolescents. *Cyberpsychology, Behavior and Social Networking*.

[19] Woods HC, Scott H (2016). #Sleepyteens: Social media use in adolescence is associated with poor sleep quality, anxiety, depression and low self-esteem. *Journal of Adolescence*.

[20] McComb CA, Vanman EJ, Tobin SJ (2023). A meta-analysis of the effects of social media exposure to upward comparison targets on self-evaluations and emotions. *Media Psychology*.

[21] Thorisdottir IE, Sigurvinsdottir R, Asgeirsdottir BB, Allegrante JP, Sigfusdottir ID (2019). Active and passive social media use and symptoms of anxiety and depressed mood among Icelandic adolescents. *Cyberpsychology, Behavior and Social Networking*.

[22] Radovic A, Gmelin T, Stein BD, Miller E (2017). Depressed adolescents’ positive and negative use of social media. *Journal of Adolescence*.

[23] Piteo EM, Ward K (2020). Review: Social networking sites and associations with depressive and anxiety symptoms in children and adolescents–a systematic review. *Child and Adolescent Mental Health*.

[24] Han DH, Kim SM, Bae S, Renshaw PF, Anderson JS (2016). A failure of suppression within the default mode network in depressed adolescents with compulsive internet game play. *Journal of Affective Disorders*.

[25] Karaer Y, Akdemir D (2019). Parenting styles, perceived social support and emotion regulation in adolescents with internet addiction. *Comprehensive Psychiatry*.

[26] Vidal C, Lhaksampa T, Miller L, Platt R (2020). Social media use and depression in adolescents: a scoping review. *International Review of Psychiatry*.

[27] Akkın Gürbüz HG, Demir T, Gökalp Özcan B, Kadak MT, Poyraz BÇ (2017). Use of social network sites among depressed adolescents. *Behaviour & Information Technology*.

[28] Colder Carras M, Aljuboori D, Shi J, Date M, Karkoub F, García Ortiz K (2024). Prevention and health promotion interventions for young people in the context of digital well-being: rapid systematic review. *Journal of Medical Internet Research*.

[29] Ünlü S, Uzun K, Arslan G (2025). Mindfulness-Based Intervention in Schools: Addressing Social Media Burnout and Enhancing Well-Being in Adolescents. *Children*.

[30] Office of the Surgeon General (OSG) (2023). Social Media and Youth Mental Health: The U.S. Surgeon General’s Advisory [Internet]. https://pubmed.ncbi.nlm.nih.gov/37721985/.

[31] McCrae N, Gettings S, Purssell E (2017). Social media and depressive symptoms in childhood and adolescence: A systematic review. *Adolescent Research Review*.

[32] Cauberghe V, Van Wesenbeeck I, De Jans S, Hudders L, Ponnet K (2021). How adolescents use social media to cope with feelings of loneliness and anxiety during COVID-19 lockdown. *Cyberpsychology, Behavior and Social Networking*.

